# Degree of Cure, Microstructures, and Properties of Carbon/Epoxy Composites Processed via Frontal Polymerization

**DOI:** 10.3390/polym16111493

**Published:** 2024-05-24

**Authors:** Aurpon Tahsin Shams, Easir Arafat Papon, Pravin S. Shinde, Jason Bara, Anwarul Haque

**Affiliations:** 1 Department of Aerospace Engineering and Mechanics, The University of Alabama, Tuscaloosa, AL 35487, USA; ashams1@crimson.ua.edu (A.T.S.); mpapon@eng.ua.edu (E.A.P.); 2 Department of Chemical and Biological Engineering, The University of Alabama, Tuscaloosa, AL 35487, USA; psshinde@ua.edu (P.S.S.); jbara@eng.ua.edu (J.B.)

**Keywords:** frontal polymerization, degree of cure, frontal velocity, front temperature, glass transition temperature, void contents

## Abstract

The frontal polymerization (FP) of carbon/epoxy (C/Ep) composites is investigated, considering FP as a viable route for the additive manufacturing (AM) of thermoset composites. Neat epoxy (Ep) resin-, short carbon fiber (SCF)-, and continuous carbon fiber (CCF)-reinforced composites are considered in this study. The evolution of the exothermic reaction temperature, polymerization frontal velocity, degree of cure, microstructures, effects of fiber concentration, fracture surface, and thermal and mechanical properties are investigated. The results show that exothermic reaction temperatures range between 110 °C and 153 °C, while the initial excitation temperatures range from 150 °C to 270 °C. It is observed that a higher fiber content increases cure time and decreases average frontal velocity, particularly in low SCF concentrations. This occurs because resin content, which predominantly drives the exothermic reaction, decreases with increased fiber content. The FP velocities of neat Ep resin- and SCF-reinforced composites are seen to be 0.58 and 0.50 mm/s, respectively. The maximum FP velocity (0.64 mm/s) is observed in CCF/Ep composites. The degree of cure (α_c_) is observed to be in the range of 70% to 85% in FP-processed composites. Such a range of α_c_ is significantly low in comparison to traditional composites processed through a long cure cycle. The glass transition temperature (T_g_) of neat epoxy resin is seen to be approximately 154 °C, and it reduces slightly to a lower value (149 °C) for SCF-reinforced composites. The microstructures show significantly high void contents (12%) and large internal cracks. These internal cracks are initiated due to high thermal residual stress developed during curing for non-uniform temperature distribution. The tensile properties of FP-cured samples are seen to be inferior in comparison to autoclave-processed neat epoxy. This occurs mostly due to the presence of large void contents, internal cracks, and a poor degree of cure. Finally, a highly efficient and controlled FP method is desirable to achieve a defect-free microstructure with improved mechanical and thermal properties.

## 1. Introduction

Fiber-reinforced thermoset composites are used as structural materials in aerospace, vehicle, marine, sports, and infrastructure applications due to their high specific strength and stiffness, light weight, fatigue, and creep resistance [1,2,3]. However, traditional thermoset composite manufacturing methods, such as hand layup, compression molding, filament winding, pultrusion, and resin transfer molding (RTM), are expensive and time-consuming due to long cure cycles and expensive equipment. In recent years, additive manufacturing (AM) has been regarded as a high-rate, efficient, and cost-effective production technology, particularly for prototype composite parts and components with complex geometries [4]. The majority of researchers used AM to produce primarily thermoplastic composite parts [5,6]. The application of additive manufacturing (AM) in the fabrication of thermoset composites is uncommon and remains in its early stages [7,8,9].

The additive manufacturing of thermoset polymers through frontal polymerization (FP) offers a promising avenue for the rapid and energy-efficient production of high-performance materials. Frontal polymerization is a method known for its rapidity, energy efficiency, and ability to produce high-performance materials. It is based on a self-sustaining process driven by the exothermic reaction of the resin system, leading to a thermal equilibrium that enables the advancement of a sharp polymerization front [10,11]. Compared to traditional autoclave-based curing methods, FP offers a quicker, more environmentally friendly, and efficient approach to polymerization [12]. Research has shown that FP allows for the production of high-performance thermosets with properties comparable to conventionally manufactured materials but with significant energy savings [13]. The self-propagating nature of FP enables the rapid curing of polymers and composites, reducing manufacturing time and energy consumption [14]. Moreover, FP has demonstrated suitability for additive manufacturing, offering advantages in energy consumption and printing speed [15]. Overall, the unique characteristics of FP position it as a superior method for the manufacturing of thermosetting polymers and composites. By utilizing techniques such as frontal ring-opening metathesis polymerization (FROMP), researchers have been able to control pot life and curing velocities to tailor the properties of thermoset polymers [16]. This method has shown potential in enabling the fabrication of complex shapes and structures, although the range of resin chemistries that can be printed using this approach is currently limited [17]. Additionally, the incorporation of cross-linking agents in FROMP processes allows for the production of thermosets with enhanced performance and tunable properties [18]. The self-sustaining nature of FP processes like FROMP enables the rapid curing of thermoset polymers and composites, offering significant time savings compared to traditional curing methods [19]. Furthermore, the use of additives and co-monomers in FP can facilitate the deconstruction of thermosets, providing avenues for recyclability and reprocess ability [20,21]. Overall, the advancements in FP techniques hold great promise for the AM of thermoset polymers with tailored properties and improved production efficiency.

Due to the short cure time and self-propagating cure reaction, FP is an appealing method that can be used in the AM of thermoset carbon/epoxy composites. This method advances polymer curing by utilizing the heat created during the exothermic reaction [22]. To activate the exothermic curing reaction, FP requires a triggering stimulus such as a heat source/UV [23]. Thermal frontal polymerization begins when a heat source and a thermal initiator are added to a monomer solution. When a photo-initiator is mixed in the monomer, a UV light can be used to commence curing. As frontal polymerization is a quick process, it can be used in repairing damages of different sizes and shapes [24]. The energy from exothermic polymerization diffuses into the surrounding regions, boosting the temperature and increasing the reaction rate [25]. The properties of cured polymers are heavily influenced by the curing agent [26,27]. Curing agents are classified as either evident or latent initiators. The monomer solution with an apparent curing agent starts the curing process without any triggering stimuli, although it takes longer to cure. The latter, on the other hand, works faster but requires UV light or a heat source as a triggering stimulus [28]. The concentration of the thermal initiator (methyl methacrylate) in the monomer affects FP velocity and temperature [29]. High FP temperatures may also exhaust latent initiators, resulting in diminished FP effects [23].

Microfibers such as carbon fiber are commonly used as reinforcing materials in thermoset composites to improve the mechanical and thermal properties like strength, stiffness, and glass transition temperature. Carbon fiber-reinforced polymer composites (CFRPs) stand out due to their superior strength-to-weight ratio, stiffness, and resistance to corrosion and fatigue when compared to metals and other synthetic fiber-reinforced composites [30]. However, the maximum mechanical properties attainable with micro-sized carbon fiber composites are significantly constrained in comparison to their continuous fiber counterparts. Mechanical properties in micron-sized fiber composites are dominated by the fiber aspect ratio, which is dependent on the critical fiber length, the minimum length required for the effective translation of fiber strength to the composite. Hence, there is a tradeoff between the maximum attainable fiber content and fiber aspect ratio. Several studies have investigated the influence of the fiber volume fraction and aspect ratio on the mechanical properties of CFRPs. It has been observed that a fiber aspect ratio of 1000 or higher is essential for producing high-performance composites, enabling them to achieve over 80% of the theoretical maximum strength and stiffness [30,31,32]. To date, there has been very little research concentrating on the FP of thermoset composites. The effects of fiber volume fractions on the frontal velocity and reaction temperature of carbon/dicylopentadiene (DCPD) thermoset composites are investigated by Luo et al. [33]. The equivalent thermal conductivity increases slowly at low fiber volume fractions but significantly at high volume fractions, impacting the heat flux distribution and potentially increasing frontal velocity [34]. For composites with high fiber volume fractions, the frontal velocity is reduced as a result of increased fiber volume fractions [35]. This is primarily owing to the lower heat sources supplied by exothermic reactions. Uitz et al. studied the reaction temperature, degree of cure, and tensile characteristics of a fused silica/resin system produced with reactive extrusion AM (REAM) [36]. The work shows that the REAM technique is feasible for short and continuous fiber-reinforced thermoset composites. Nimbagal et al. and Bomze examined the FP of short carbon fibers and carbon nanofiber (CNF) [37,38]. The use of short carbon fiber and the CNF combinations lowers polymer oxidation, resulting in better mechanical characteristics in comparison to CNF-reinforced composites [37]. When utilized as catalysts, carbon-based compounds such as CNF and graphene oxide can aid the FP process [39]. This carbon-based substance can lower the curing reaction temperature, reducing the probability of thermal initiator burnout [40]. As a result, they can minimize activation energy and accelerate the initial polymerization reaction [41].

It is obvious that the short cure cycle of FP makes it a novel process for the AM of composites. This paper primarily focuses on studying the effects of short carbon fiber reinforcements in FP and analyzing void contents, tensile properties, fracture behavior, and thermal properties such as the glass transition temperature (T_g_) and degree of cure (α_c_) experimentally which are rarely observed in the open literature. Such observations will facilitate identifying and improving the shortcomings of FP to apply it in the AM of thermoset composites.

## 2. Experimental Work

### 2.1. Materials

Neat epoxy resin (EPON 828) containing bisphenol A/epichlorohydrin was purchased from Hexion (Columbus, OH, USA). Thermal initiator 1,1,2,2-tetraphenyl-1,2-ethanediol (TPED) and cationic initiator, bis[4-(tert-butyl) phenyl] iodonium Tetra(nonafluoro-tertbutoxy) aluminate, were received from Sigma-Aldrich (Burlington, MA, USA) and TCI America (Tokyo, Japan), respectively. Short carbon fibers (PX35 milled fibers) were procured from Zoltek Supplies (St. Louis, MO, USA). These were PAN (polyacrylonitrile)-based round fibers, and their purity was 99%. The density, diameter, and average length of the short carbon fiber (SCF) were 1.75 g/cc, 7.2 µm, and 100 µm, respectively. The continuous carbon fiber (CCF) used in this work was a tow consisting of approximately six thousand single filaments.

### 2.2. Mixture Preparation

Two mixtures were prepared for the frontal polymerization of SCF/Ep and CCF/Ep thermoset composites. Type-1 mixture (M-1) was prepared by mixing 1%, 2%, 3% SCF by weight with neat epoxy resin in a rotating cup using a mechanical stirrer; then, thermal initiator (TI-3%) and cationic initiator (CI-1%) were added and mixed into the SCF/Ep mixture. Finally, the rotating cup with the M-1 mixture was placed in a Thinky (Tokyo, Japan) planetary centrifugal mixer. The Thinky mixer uses centrifugal and high shear forces to mix micro-sized particles and nanomaterials in viscous resin without bubble formation. The M-1 mixture was rotated for six minutes at 200 RPM to make a well-dispersed SCF/Ep composite mixture. Type-2 mixture (M-2) was prepared similarly without incorporating SCF reinforcing materials. This M-2 mixture was used to make cured neat epoxy and CCF/Ep composites using the FP method.

### 2.3. Specimen Preparation

**Figure 1:**
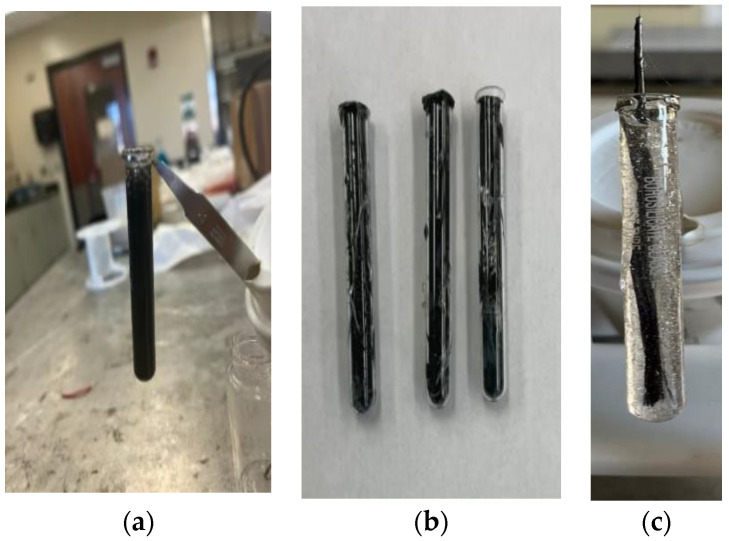
Specimen preparation using FP. (**a**) SCF/Ep mixture, (**b**) cured SCF/Ep composites, (**c**) CCF/Ep mixture.

The SCF/Ep mixture (M-1) was poured into a glass test tube (length/diameter: 75 mm × 10 mm) using a syringe to study frontal polymerization. Test tubes with (a) uncured SCF/Ep composite mixture (M-1), (b) cured SCF/Ep composites, and (c) single continuous carbon fiber tow in uncured epoxy resin mixture (M-2) are shown in Figure 1. Neat epoxy resin, thermal, and cationic initiator mixture (M-3) was poured into a test tube to carry out FP and make cured neat epoxy. An external heat source from a hot plate was incorporated at the bottom of the test tube to trigger the exothermic reaction of FP. The initiation temperature of the hot plate was calibrated using an IR camera. Once the reaction initiated and proceeded, the test tube was removed from the triggering heat source. As the frontal polymerization continued, the temperature distribution and the path were monitored using an IR camera. The time was recorded as the reaction proceeded along the test tube length. Frontal velocity was calculated from the time and travel path.

### 2.4. Tensile Test

Tensile testing was carried out in a Q-test machine under displacement control at a rate of 0.127 mm/s (0.005 in/s) with a 25 KN load cell using a V-shaped grip to hold the specimens, and load/displacement data were acquired through the data acquisition system. The specimen geometry of the sample is length 75 mm (2.952 inches) and diameter 10 mm. The tensile strength, modulus, and failure strain were determined from stress/strain plots.

### 2.5. Microstructural Examination

The microstructural examinations of all the samples were carried out using a digital optical microscope and scanning electron microscope. The microstructure samples for the optical microscope were cut diagonally from the cured specimen using a diamond cutter. Then, the surface was prepared through sequential grinding and polishing. Sandpapers of different grit sizes 480 and 800 were used to prepare the surface for microscopic observation. Final polishing was conducted using a polishing cloth to make the surface smooth. Finally, the surface was cleaned using alumina suspension to remove all the debris. The specimen was mounted on a microscopic slide to quantify void contents and cracks. An image processing software “Image J 1.53t” was used to measure void contents.

A scanning electron microscope was used to study the topography of fractured surfaces. An ETD detector was used to capture secondary electrons generated from the fractured surface to describe the topography. As epoxy was a nonconductive material, a thin gold coating was used to make it conductive. A low current was used to obtain a representative low-magnitude image of the surface. The working distance was kept at 10 mm. The beam voltage varied between 12 KV and 20 KV.

### 2.6. Differential Scanning Calorimetry (DSC)

A DSC instrument was used to determine the glass transition temperature (T_g_) and degree of cure (α_c_) of neat epoxy resin and SCF/Ep composites. DSC defines T_g_ as a change in heat capacity as the polymer matrix converts from the glassy state to the rubbery state. T_g_ was calculated using a half-height method in which the midpoint is the Y-axis value, halfway between the calculated onset and calculated end set of the glass transition region. The temperature corresponding to that Y-axis value was reported as T_g_.

The degree of cure for each sample was calculated using the following equation.

Degree of cure = 1 − (H/H_o_)

H = total heat of reaction for the partially cured samples
H_o_ = total heat of reaction for the uncured samples

The uncured samples for the DSC experiment were prepared in the same way as the sample’s preparation described in Mixture Preparation (Section 2.2). The liquid mixture was placed in a DSC pan and heated to determine the total heat of reaction. A small chunk was cut from the partially cured samples to measure the number of uncured monomers in it, and a powdered form of the chunk was placed on the DSC pan to determine the T_g_ of the sample. The measurement was performed at the 0 °C to 300 °C range. The heating rate was 3 °C/min. The T_g_ measurement was conducted from 30 °C to 300 °C, and the heating rate was 50 °C/min.

## 3. Reaction Kinetics

**Figure 2:**
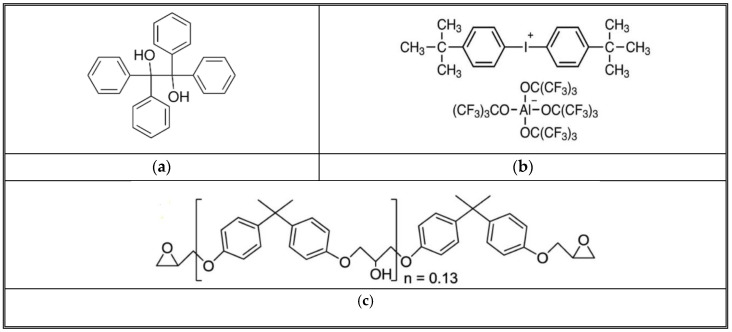
Structure of TPED, cationic initiator, and Epon 828. (**a**) TPED-C_26_H_22_O_2_ (**b**) Bis [4-(tert-butyl) phenyl iodonium Tetra (nonafluoro-tertbutoxy) aluminate (IOC-A) (**c**) DGEBA (diglycidyl ether of bisphenol A or Epon 828).

The frontal polymerization (FP) process, especially in the context of epoxy resins, is initiated and propagated through a series of chemical reactions that the initiators catalyze. The thermal initiator (TPED) and the cationic initiator are shown in Figure 2. The structure of TPED, the cationic initiator, and Epon 828 work together to create conditions favorable for polymerization but in different roles [42]. TPED, serving as the thermal initiator, is primarily involved in generating the reactive conditions necessary for the cationic initiator to activate [43]. It may not directly interact with the monomers to break them and form polymers. Instead, its role is to facilitate the formation of active sites (cationic species) by the cationic initiator through thermal decomposition or by forming a charge transfer complex that helps to activate the initiator [44]. Upon activation facilitated by the thermal decomposition of TPED, the cationic initiator generates a cationic species that can interact with the epoxy monomer shown in Figure 2 [45,46]. This interaction typically involves the opening of the epoxide ring of the monomer, allowing it to react with other monomer molecules or growing polymer chains. This is the key step where the monomer is “broken” (or, more accurately, activated and reacted) to form the polymer [47]. The cationic species effectively acts as the catalyst that initiates the polymerization by opening the monomer’s epoxide ring, enabling the linking of monomer units into a polymer chain [38]. Thus, breaking the monomer chain generates the heat to decompose the TPED further.

**Figure 3:**
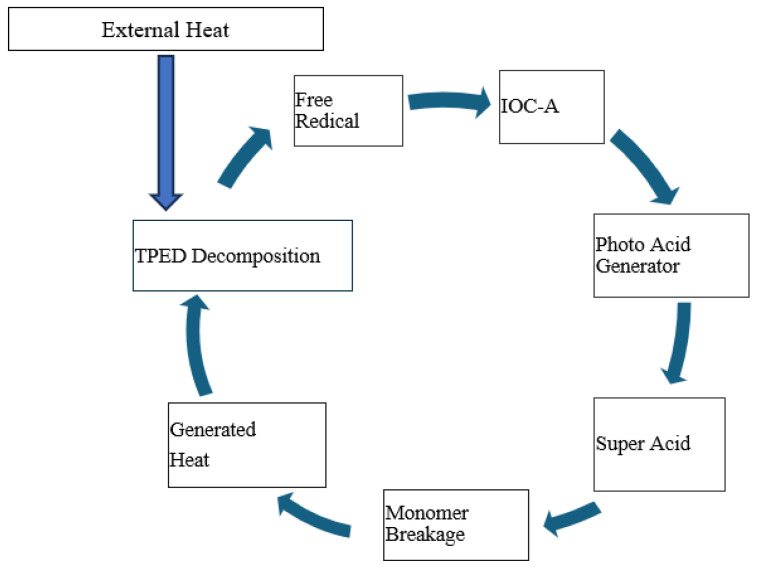
Schematic of reaction kinetics in frontal polymerization process.

Figure 3 shows a schematic of reaction kinetics that occur in FP. Polymerization in this DGEBE system is triggered by heat, which initiates the decomposition of the thermal initiator, TPED. Being an effective electron donor, TPED releases electrons upon exposure to initial or external heat, resulting in its positive charge. This positively charged TPED then activates IOC-A, which, in turn, accepts an electron to become a negatively charged photo acid generator (PAG). As a potent Bronsted acid, the PAG is capable of breaking the bonds in epoxy monomers, leading to the formation of epoxy polymers. This breaking process releases heat, which further decomposes TPED, thereby perpetuating the cycle. Consequently, this self-sustaining mechanism allows for the rapid and autonomous progression of the polymerization process. In the case of carbon/epoxy, heat and the cationic initiator act in the same manner, but the frontal velocity and the reaction temperatures vary depending upon initiation temperature and carbon contents.

## 4. Results and Discussion

### 4.1. Temperature Distribution

**Figure 4:**
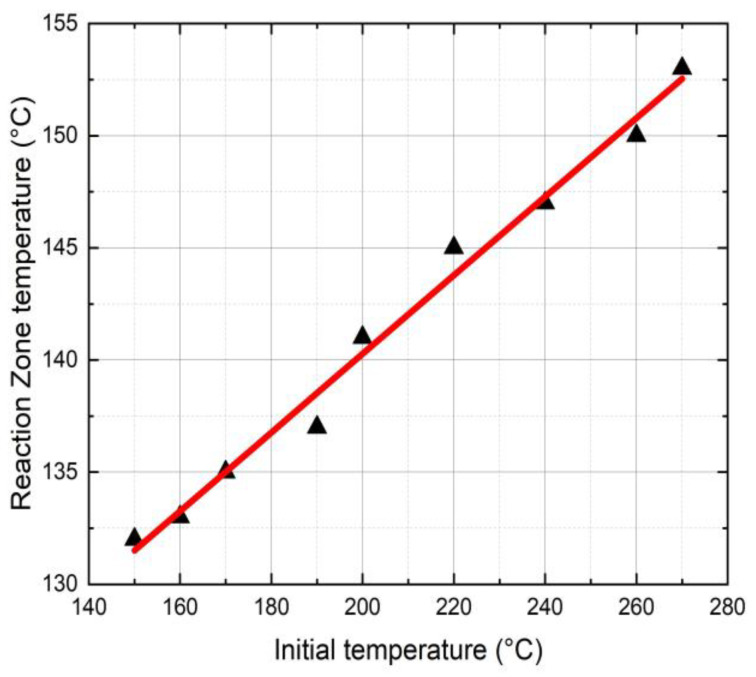
Initial temperature vs. reaction zone temperature in neat epoxy.

Figure 4 shows the effects of the initial triggering temperature (T_i_) on the FP reaction zone temperature. In this study, T_i_ varied from 150 °C to 270 °C resulting in a reaction temperature (T_r_) from 133 °C to 155 °C. The reaction zone temperature is an essential parameter in FP as it determines the stability of the initiator and the polymer conversion. It is to be noted that a higher reaction zone temperature may lead to initiator burnout resulting in reduced FP velocity.

**Figure 5:**
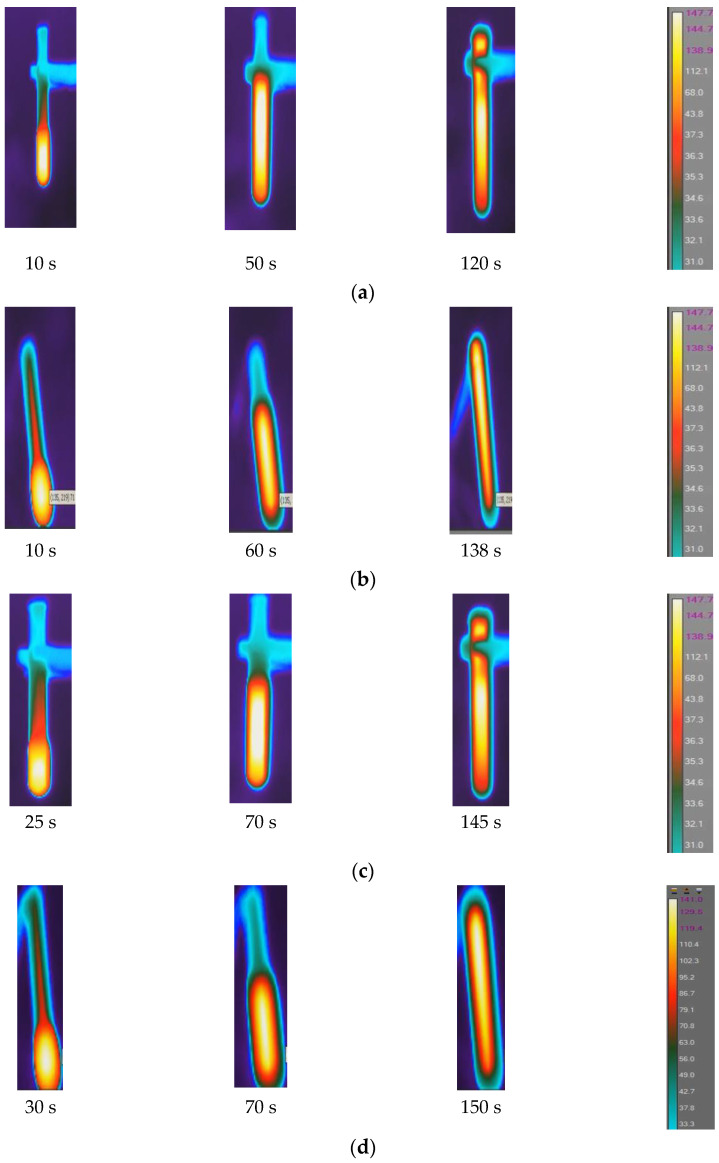

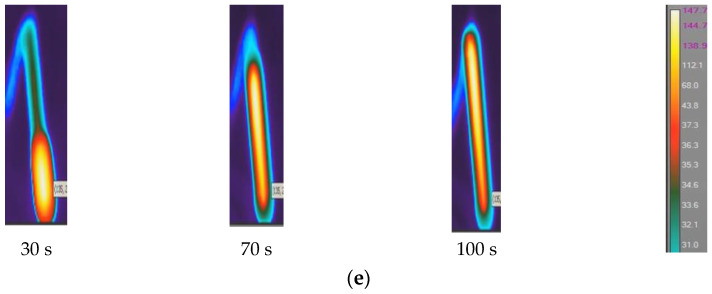
Temperature distribution in FP reaction zone: (**a**) neat epoxy resin, (**b**) epoxy with 1% SCF, (**c**) epoxy with 2% SCF, (**d**) epoxy with 3% SCF, and (**e**) epoxy with continuous fiber.

Figure 5 shows IR images displaying temperature distribution surrounding the reaction zones and path at different time intervals for neat resin, SCF/Ep composites, and CCF/Ep composites. The temperature profile has three stages due to heat wave propagation such as steady (no polymerization), rising (front/heat wave coming), and fall (front/heat wave departing). In the steady state, no polymerization occurs since no temperature changes occur in the reaction front. Thermal diffusion raises temperatures as the front approaches. Due to intense exothermic reaction, the narrow front reaction zone’s temperature increases fast to T_max_ during the rising state. Thermal diffusion lowers the temperature when the front leaves during the fall stage [48].

The initial triggering temperature was set at 240 °C. In each case, the maximum temperature is observed at the central region of the reaction zone. The maximum reaction temperature (T_r_) in neat epoxy is observed to be 147 °C, whereas the same in SCF/Ep and CCF/EP is seen to be 140 °C and 135 °C. Increased fiber concentrations reduce resin contents in the system, eventually influencing reaction temperature. The fibers in the resin hindered the generation of heat. It is evident in Figure 5a that FP initiated after 10 s and eventually continued until 120 s. The time to complete polymerization in SCF/Ep composites (ranges from 138 s to 150 s) is higher than neat epoxy resin. In Figure 5b,c,e, the reaction time is further extended with increased SCF concentrations. Figure 5e depicts the reaction time of the composites (100 s) using a single carbon fiber tow. This duration is less than that of SCF/Ep composites but close to that of neat resin. The reason for such an analogy is likely due to neat resin surrounding a single carbon fiber tow, which ensures uninterrupted heat flow that fuels FP. In the case of continuous fibers, the temperature distribution is seen to be uniform, but the T_max_ is observed to be the smallest. This is because the generated exothermic heat can travel through the bundle of fibers instead of accumulating in localized regions and increasing temperature.

### 4.2. Frontal Velocity

**Figure 6:**
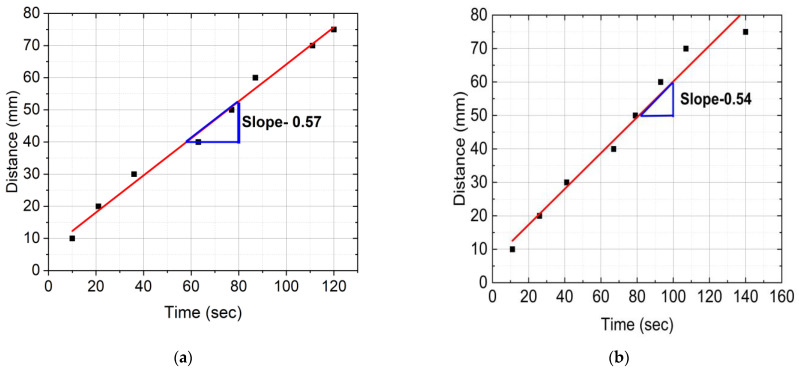

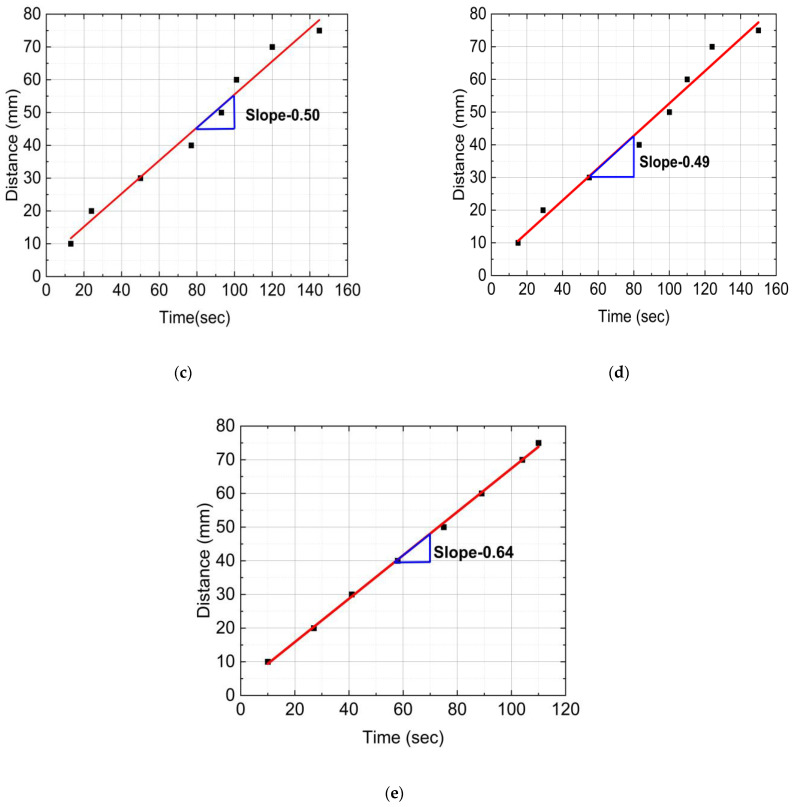
Distance verses time plots of FP reactions: (**a**) neat epoxy, (**b**) epoxy with 1% SCF, (**c**) epoxy with 2% SCF, (**d**) epoxy with 3% SCF, and (**e**) epoxy with continuous fiber.

Figure 6 provides distance versus time plots of FP reactions for neat epoxy resin, SCF/Ep composites, and CCF/Ep composites. These data were captured from the IR images. The time to reach a marked distance in the test tube was recorded by a stopwatch. First, every 10 mm, the test tube was marked using a permanent marker. Then, the time was recorded when the polymerization head was reached in each marked point (every 10 mm). The reaction’s frontal velocity (Vf) plays as an essential parameter in the polymerization process. It was calculated from the slope of a linear plot generated from the data points shown in Figure 6.

Figure 6a,e shows the highest V_f_ (0.64 mm/s) in CCF/composites and then 0.57 mm/s in neat epoxy resin. V_f_ in SCF/Ep composites with 1%, 2%, and 3% is seen to be 0.54, 0.50, and 0.49 mm/s, respectively, as shown in Figure 6b–d. The result shows a reducing trend in V_f_ with increased SCF concentrations. It is to be noted that SCFs can increase the viscosity of the epoxy matrix, slowing down the polymerization reaction. This viscosity increase reduces reactive species’ mobility, making the polymerization reaction harder to spread through the material. This increase in viscosity might slow down the reaction kinetics, resulting in a lower frontal velocity. Thus, SCF can reduce heat transfer efficiency, decreasing the reaction rate and frontal velocity. Moreover, SCF tends to agglomerate, which acts as a barrier to heat transfer. The intense interaction between the SCF and epoxy can reduce the mobility of the monomers. This contributes to reduced polymerization. Although adding SCF increases the conductivity of the mixer, the results show reduced FP velocity decreases. As a result, the conductivity of the mixer plays a less critical role in this study.

The rate of frontal epoxy polymerization might vary for several reasons. These include material properties, temperature gradients, heat transport limitations, concentration gradients, heterogeneous nucleation, and stress-induced polymerization. Exothermic heat generation results in temperature gradients that change the kinetics and viscosity of reactions. Heat dissipation may be hampered in some areas by restrictions on heat transport. Concentration gradients influence the kinetics of polymerization and reactions. Localized response rates are a result of heterogeneous nucleation. Polymerization modifies molecular mobility and kinetics, affecting velocity. Viscosity and filler dispersion also influence the frontal velocity. As a result, variations in V_f_ are also influenced by other factors resulting from increased SCF concentrations.

### 4.3. Void Contents

**Figure 7:**
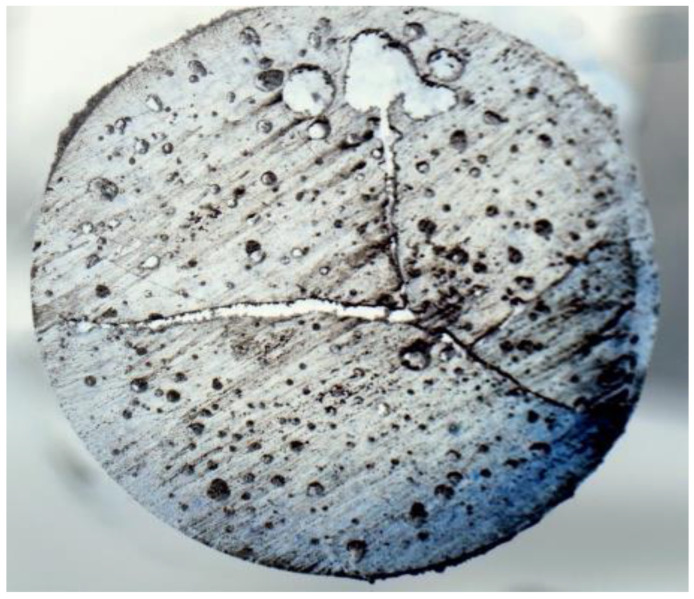
Microscopic cross-sectional view (7.5×) of pure epoxy.

Figure 7 shows the microstructure of cured neat epoxy. The micrographs show the presence of voids and cracks initiated from the central region. In general, voids are formed mostly due to the formation of gases during the curing process. The presence of voids in mixtures before curing is also not desirable for achieving a cured sample without voids. As a result, the efficiency of mixture preparation without voids plays an important role in specimen preparation. The efficiency of the specimen preparation may be enhanced by pouring mixtures into test tubes directly using a funnel instead of a syringe. Suction by a syringe may result in bubble formation into the mixture. Mixtures with bubbles in test tubes before curing may result in enhanced void contents in cured samples. The cracks that are initiated at the central region and then propagated toward the periphery are possibly because of the residual stress generated due to the temperature gradient. The insulation of the glass tube might minimize such a temperature gradient and minimize crack propagation. During the curing process, the covalent bond replaces the secondary van der Waals bond, causing chemical shrinkage in the resin and developing residual stress. Moreover, polymerization in a confined test tube (illustrated in Figure 1) restricts the thermal expansion of the epoxy. The measured void content is almost 12% by volume, which is comparatively high compared to conventional techniques such as autoclave and vacuum bagging. The presence of such a high void content and internal cracks due to residual stress significantly degrades the mechanical properties of FP-processed composites.

### 4.4. Tensile Properties

**Figure 8:**
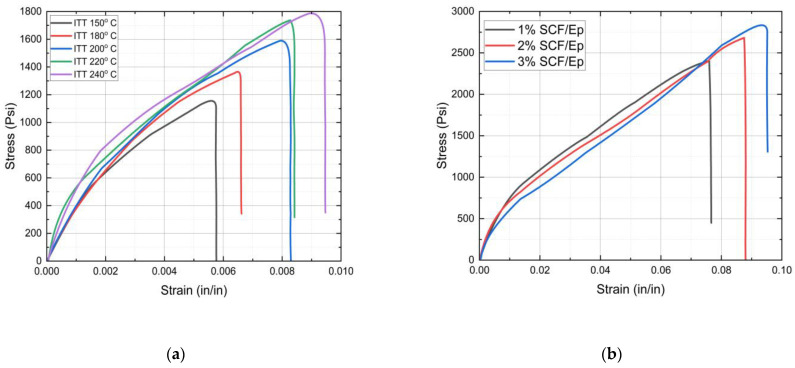
Stress *vs*. strain plots: (**a**) neat epoxy at different ITTs, (**b**) SCF/Ep composites at different SCF percentages.

The initial triggering temperature (ITT) is an essential factor in frontal polymerization, which influences the curing reaction of the resin and, eventually, the microstructures. Reaction kinetics and curing at a higher initial temperature may increase tensile strength. But the higher initial temperature can also lead to thermal degradation, thus reducing the tensile strength. The effects of the initial triggering temperature on the stress/strain plots of neat epoxy resin are shown in Figure 8a. Table 1 provides tensile failure stress and failure strain data for neat epoxy resin at different initial triggering temperatures. It appears that both failure strength and failure strain were enhanced with an increased initial triggering temperature.

The results showed the highest tensile strength for the specimens when the triggering temperature was set at 240 °C. The nature of the stress/strain plots for neat epoxy resin is seen to be primarily nonlinear. The tensile strength is seen to be significantly low in comparison to epoxy cured through conventional methods. The reasons for such low tensile strength are possibly due to large void contents (12%) and a poor degree of cure (85%) that are observed in neat epoxy.

Figure 8b shows stress/strain plots of SCF/Ep composites with 1%, 2%, and 3% SCF concentrations. Table 2 provides the failure stress and failure strain of SCF epoxy composites with 1 to 3% fiber content. The results show enhanced tensile strength in SCF/Ep composites in comparison to neat epoxy resin. It appears that both failure strength and failure strain increased with enhanced short carbon fiber contents within a range from 1% to 3%. Such an enhancement of both failure strength and failure strain was also observed in TiO_2_/epoxy nanocomposites in comparison to neat resin [49].

Although, tensile testing was not carried out with the CCF/Ep composite due to the unavailability of a proper gripping fixture. But a similar enhancement in tensile strength is expected in the CCF/Ep composite. Such an enhancement in properties indicates good bonding between SCF and epoxy resin. The evidence of a good interfacial bonding, fiber pullout, and uniform dispersion of SCF was observed in SEM micrographs. The highest tensile strength is observed to be in SCF/Ep composites with 3% SCF. The nature of the plots shows linear behavior at the early stage and then gradually becomes nonlinear.

### 4.5. SEM of the Fractured Surface

**Figure 9:**
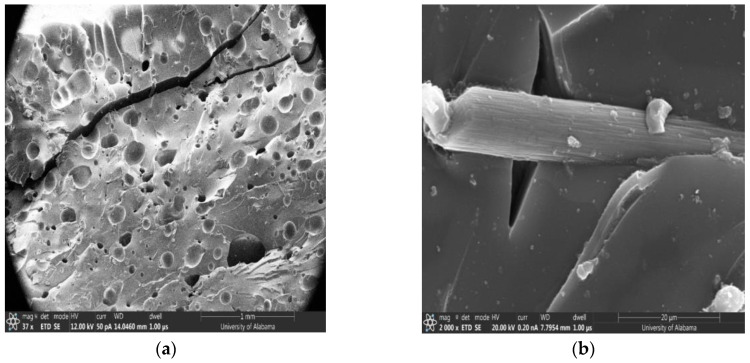

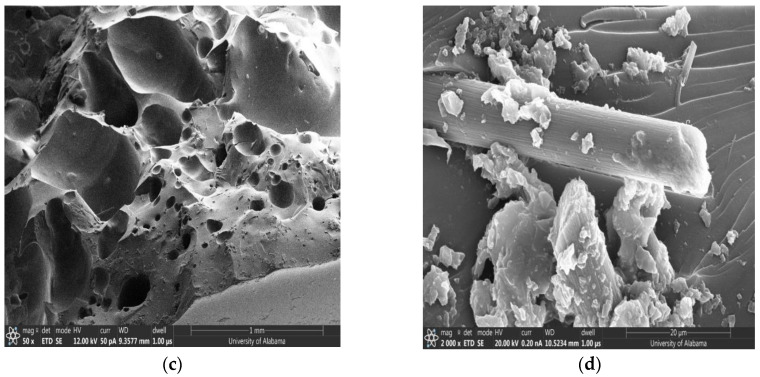
SEM image of fractured SCF/Ep: (**a**) large void contents and matrix cracking, (**b**) fiber pullout, debonding, and matrix cracking, (**c**) SCF dispersion, and (**d**) fiber pullout and matrix on fiber surface.

Figure 9a shows a fractured surface of SCF/Ep composites, indicating the presence of large void contents and matrix cracking. The mechanical performance of FP-cured carbon/epoxy composites is intricately linked to their internal microstructure, particularly the presence and distribution of voids and cracks, and the quality of interfacial bonding between the fibers and the matrix. Optimizing the curing process to reduce void content, minimize temperature gradients, and enhance fiber matrix bonding is crucial for improving the mechanical properties of these composites. Each of these factors must be carefully controlled and optimized to harness the full potential of FP-cured composites in high-performance applications. Further studies and improvements in curing techniques and composite formulations could help mitigate these issues, leading to stronger, more reliable materials. Detailed observations also show SCF random distribution without agglomeration and fiber pullout within epoxy resin. The large matrix cracking possibly resulted from residual stress during curing. Such large matrix cracking was also observed in the unloaded neat epoxy specimen, as shown in Figure 9. The fractured surface in Figure 9a shows undulation, indicating significant energy absorption during the fracture process.

In Figure 9b, a higher magnification micrograph of SCF/Ep composites clearly shows a single fiber with debonding, matrix cracking, and the fiber pullout mechanism. It is seen that there are some chunks of epoxy still attached to the SCF’s surface. But in general, the fiber surface is seen to be very smooth, indicating poor interfacial bonding.

Figure 9b shows random dispersion of SCF without agglomeration. A large number of void contents and undulation were also observed in the fractured surface. Figure 9d clearly shows fiber pullout with epoxy resin attached to the surface of the SCF. This shows good bonding indicating enhanced strength in SCF/Ep composites in comparison to neat resin.

### 4.6. Glass Transition Temperature and Degree of Cure

**Figure 10:**
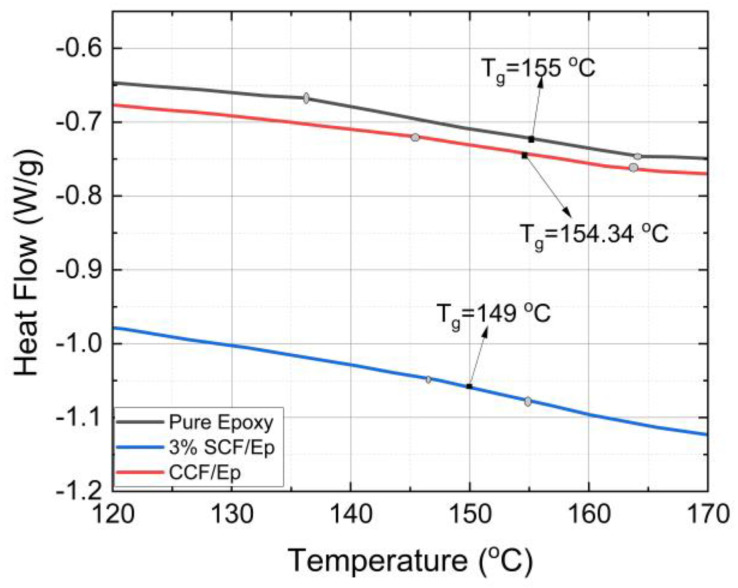
Heat flow vs. temperature plots of epoxy with 3% SCF, epoxy with continuous fiber.

The T_g_ and α_c_ of FP-cured neat epoxy resin and SCF/Ep composites were determined from the heat flow vs. temperature plots obtained from the differential scanning calorimetry (DSC) experiments. Table 3 shows the heat flow vs. temperature plots of powdered cured neat epoxy, 3%-SCF/Ep and CCF/Ep composites. The method to determine T_g_ using a DSC is described earlier. Figure 10 shows the T_g_ of neat epoxy (155 °C) and SCF/Ep with 1%, 2%, and 3% of SCF concentrations. The T_g_ of neat epoxy is seen to be comparatively higher than SCF/Ep composites. The effects of SCF weight concentration within a range of 1–3% show insignificant variations in the T_g_ of SCF/Ep composites. As a result, only one such plot is shown in Figure 10. The T_g_ of continuous CCF/Ep composites is seen to be 154.34 °C which is almost same as neat epoxy. The addition of short carbon fibers (SCFs), which leads to a slightly lower T_g_ of 149 °C, can be attributed to the interaction between the SCFs and the epoxy matrix. These interactions can disrupt the polymer structure, making it slightly more flexible and lowering its T_g_. In contrast, the T_g_ of epoxy with continuous fiber is observed to be almost the same as neat resin. Such a trend is most likely due to less interaction in the neat epoxy polymer structure.

**Figure 11:**
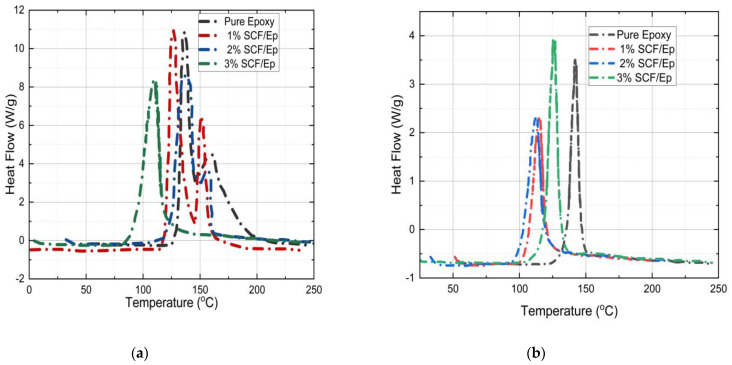
Temperature (°C) vs. heat flow (Wg^−1^) for (**a**) uncured samples. (**b**) Already cured sample.

The heat flow vs. temperature plots of both uncured and partially cured pure epoxy and SCF/Ep are shown in Figure 11a,b in order to determine the degree of cure (α_c_). It is known that the area under the peak is the total heat of reaction [50]. The heating rate in the test was kept uniform at 3 °C/min. The total heat flow is not seen to be changed with the heating rate. Table 3 shows total heat flow for initially uncured neat epoxy (278 J/g) resin which is observed to be reduced with the inclusion of SCF. The heat flow for 1%, 2%, and 3% SCF /Ep composites is seen to be 230 J/g, 219 J/g, and 189 J/g, respectively. In the case of cured samples, the heat of reaction for pure epoxy, epoxy with 1%, 2%, and 3% SCF is seen to be 42 J/g, 39 J/g, 45 J/g, and 50 J/g, respectively. These magnitudes are comparatively low as they correspond to uncured resin that remains in FP-processed samples. The degree of cure for pure epoxy and 1%, 2%, and 3% SCF/Ep composites is observed to be 85%, 81%, 75%, and 71%, respectively. These are calculated using the equation provided earlier. The degree of cure reduces with the increase in the SCF percentage. The degree of cure is also seen to be higher in pure epoxy.

In summary, the change in Tg with the incorporation SCF is indicative of altered mechanical and thermal properties. A lower Tg suggests increased flexibility and reduced thermal stability, while a higher Tg implies enhanced rigidity and thermal resistance. Understanding these changes is crucial for tailoring polymer materials for specific applications, particularly in environments with varying thermal conditions. The degree of cure (α_c_) of all the FP cured samples is low in comparison to composites cured in a traditional manufacturing process due to the inferior cross-linking density. This resulted in poor mechanical properties of the FP-cured samples. Further studies are required to increase the degree of cure and to minimize void contents in FP-cured samples.

## 5. Conclusions

The following conclusions are drawn from the results of this work.

1. The initial triggering temperature influences the FP reaction temperature and mechanical properties of neat epoxy resin. A triggering temperature of 270 °C shows a maximum reaction temperature of 153 °C.
2. The amount of SCF contents influences reaction temperature, glass transition temperature, total heat generation, degree of cure, frontal velocity, and mechanical properties of composites. The glass transition temperature and degree of cure in neat resin are seen to be higher in comparison to SCF/Ep composites. At smaller SCF loadings in SCF/Ep composites, the reduction in epoxy content leads to lower reaction zone temperatures and slower frontal velocities.
3. The frontal velocity in CCF/Ep composites is seen to be higher in comparison to SCF/Ep and pure epoxy. This is because of uninterrupted exothermic reaction in resin.
4. The tensile strength in neat epoxy (FP-cured) is seen to be comparatively inferior to the same cured by the conventional autoclave method. This is mostly due to large void contents, internal cracks, and a poor degree of cure in FP samples. An efficient FP sample preparation method is highly desirable to achieve improved microstructures and mechanical properties.

## Figures and Tables

**Table 1:** Effects of initial triggering temperature in failure stress and failure strain of neat epoxy.

Initiation Temperature	150 °C	200 °C	240 °C
Failure strain (in/in)	0.0061 ± 0.000377	0.0079 ± 0.000432	0.0093 ± 0.000113
Failure stress (PSI)	1130 ± 30.81	1521 ± 88.99	1701 ± 73.54

**Table 2:** Effects of SCF percentage in failure stress and failure strain SCF/Ep composite.

SCF Percentage	1%	2%	3%
Failure strain (in/in)	0.07509 ± 0.000643	0.08117 ± 0.004566	0.084 ± 0.007257
Failure stress (PSI)	2381 ± 15.30	2520 ± 122.01	2782 ± 116.67

**Table 3:** Heat of reactions, glass transition temperature (T_g_), and degree of cure (α_c_) of epoxy and SCF/Ep composites.

Sample Type	Pure Epoxy	1% SCF/Ep	2% SCF/Ep	3% SCF/Ep
The area under the curve (ΔH) for uncured resin	278 J/g	230 J/g	219 J/g	189 J/g
The area under the curve (ΔH) for cured resin	42 J/g	39 J/g	45 J/g	50 J/g
Degree of cure (α_c_)	85%	83%	79%	73%
T_g_	155 °C	149 °C	148 °C	149 °C

## Data Availability

Data are contained within the article.
